# Patterns of Drug Utilization and Self-Medication Practices: A Cross Sectional Study

**DOI:** 10.3390/pharmacy11060183

**Published:** 2023-11-22

**Authors:** Hamod Al-Omrani, Mandeep Kaur Marwah, Razan Al-Whaib, Mohammed Mekkawy, Hala Shokr

**Affiliations:** 1 Pharmacy Practice Department, College of Clinical Pharmacy, King Faisal University, Al-Ahsaa 31982, Saudi Arabia; 2 Aston Medical School, College of Health and Life Sciences, Aston University, Birmingham B4 7ET, UK; 3 High Institute of Public Health, Alexandria University, Alexandria 21526, Egypt; 4 Pharmacy Division, Faculty of Biology, Medicine and Health, The University of Manchester, Manchester M13 9PL, UK

**Keywords:** self-medication, drug utilization, over the counter drugs, drug misuse

## Abstract

Background: Self-medication (SM) is a growing phenomenon worldwide that has recently been classified as one of the most serious public health problems. SM can result in an incorrect self-diagnosis, inappropriate treatment, potential adverse reactions, interactions, and the masking of more sinister disease. Objectives: To assess the prevalence of SM practices amongst healthcare professionals and the general population in Saudi Arabia and to identify the sociodemographic contributing factors to this practice. Methods: A total of 540 participants were enrolled in this study. Participants were asked to complete the study questionnaire comprising two sections to gather demographic information and to collect data regarding SM practice. Results: The prevalence of SM practice among the study participants was 78.6%, and it was the highest among the middle age groups of (21–40 years) 82% compared to the younger and older age groups. The observed prevalence was higher in the female participants (53.7%) and those who live alone. Type of education did not affect the attitude of SM (*p* = 0.374); however, level of education strongly affected the prevalence of the SM, with higher incidence among university graduates (*p* < 0.001). Analgesics with antipyretics properties were the most self-medicated drugs. Patients considering their illnesses minor was the main reason behind the high prevalence of this practice. Among healthcare professions the highest prevalence of SM was found among pharmacists (95%), followed by physicians, nurses, and other medical practitioners. Conclusion: The general population should be better educated and made conscious about the consequences, risks, and side effects of SM. Awareness campaigns may prove to be useful in this matter, allowing the patient to consider the appropriateness of this practice. Individuals in the age group of 21–40 years, females, and those who live alone should be considered priority target populations in the design and implementation of SM awareness projects.

## 1. Introduction

Self-medication (SM) has gained significant importance in the healthcare sector over the past decade, with its definition constantly evolving to keep pace with the rapid changes in international drug and safety regulations [1]. The initial definition of SM, introduced by the World Health Organization (WHO), referred to the use of pharmaceutical products to address undiagnosed or self-diagnosed symptoms [2]. However, as over-the-counter medications (OTC) proliferated and direct-to-consumer advertisements (DTCA) became widespread, the definition expanded to encompass the consumption of prescribed drugs for chronic or recurrent illnesses without physician referral [3,4,5]. Later, the definition was further broadened to incorporate the utilization of herbal/traditional medicine and homemade remedies without medical supervision [6,7]. SM also encompasses patients’ choice of medication for managing a chronic or recurring condition following the initial diagnosis and prescription by a physician, as well as the act of administering medicinal products to family members, particularly infants, children, or the elderly [8].

Over the past decade, the widespread availability, accessibility, and affordability of drugs have contributed to the emergence of SM as a significant global public health issue [9,10]. Recent studies have indicated a consistent and upward trend in this practice in recent years, with estimates from various researchers suggesting that nearly 40% of individuals experiencing new medical complaints resort to non-prescribed drugs to alleviate their symptoms [11,12]. Although it is important to note that SM should be distinguished from responsible self-care, where individuals take appropriate measures for their well-being under the guidance of healthcare professionals, this rapid expansion of SM has resulted in approximately 70% of the global disease burden [13]. Moreover, SM has been directly associated with numerous critical drug-related issues, including the rise of antibiotic resistance [14], drug toxicities, misuse, abuse [15], polypharmacy [16], drug–drug interactions [17,18], and even drug-induced congenital anomalies [19]. Another key problem associated with this practice is misdiagnosis. Without proper medical assessment and diagnosis, individuals may inaccurately identify their health conditions, leading to the use of inappropriate medications. This can result in delayed or ineffective treatment, potentially exacerbating the underlying issue [20]. Moreover, SM can mask underlying medical conditions. By merely addressing the symptoms without proper diagnosis, individuals may unknowingly delay necessary medical attention, allowing potentially serious illnesses to progress unchecked [21].

The relationship between SM and the economy of countries is complex and multifaceted. Initially, it was believed that SM is primarily prevalent in economically deprived countries and developing nations, where health literacy, fewer restrictions on the sale of drugs, limited healthcare resources, and high healthcare costs can make it difficult for individuals to seek medical attention for minor health issues [22,23,24]. However, recent research has shown a significant increase in the percentage of drugs being sold and used without a prescription even in developed countries [25,26]. For instance, in Germany, it has been reported that 40% of the population engages in SM at least once a month [27]. Similarly, in Europe, the prevalence of SM ranges from 7% to 50% [28], with the highest rates observed in the less economically developed countries in northwest Europe [29,30].

Reports indicate that in the United States, approximately 40% of the population self-medicates without consulting a medical professional [31], similar patterns were reported in Finland with rates ranging from 17% to 39% [32,33], while in Indonesia, the practice has surged to as high as 90% [34]. This SM trend extends also to the gulf region, where individuals often rely on over-the-counter medications to address minor health concerns [35]. In the kingdom of Saudi Arabia (KSA), researchers have reported that as many as 81% of the general population have acknowledged using medications without a prescription at some point in their lives [35]. In addition to over-the-counter drugs, many people in the Gulf region turn to age-old folk remedies and traditional healing practices to alleviate common ailments [36]. These folk remedies often involve natural ingredients, herbal treatments, or homeopathic solutions that have been passed down through generations. The combination of over-the-counter medications and traditional folk remedies reflects the diverse and multifaceted approach to self-medication in this region. It is important to note that while some of these folk remedies may have cultural significance and can provide relief for minor ailments, their safety and effectiveness should be evaluated, and individuals should exercise caution when using them, especially for more serious health conditions.

At present, the World Health Organization (WHO) advocates for SM in cases of minor illnesses as a strategy to alleviate the growing burden on healthcare services, particularly in countries with limited resources [37]. However, the prominent barrier to promoting this practice lies in the potential misuse of medications by patients. Recognizing the significance of these concerns surrounding SM, the primary objective of this study is to investigate several aspects: the prevalence of SM practices among both healthcare professionals and the general population, the key sociodemographic factors influencing such practices, and the associated risks within the KSA.

## 2. Materials and Methods

### 2.1. Study Population

In this cross-sectional study utilizing descriptive questionnaires, participants were recruited from diverse regions across KSA, encompassing both healthcare professionals and the general population from 2017 to 2020.

A semi-structured questionnaire consisting of open- and close-ended questions was used to gather relevant information regarding the study variables. The questionnaire was divided into two sections: Section (A) focused on demographic information such as age, gender, and level of education; Section (B) included questions related to participants’ attitudes, perceptions, and practices of SM. Before starting the evaluation, participants were provided with detailed explanations of the study purpose. The questionnaires were distributed by the primary researcher who also provided clear definitions of certain terms to ensure consistency among participants for the purpose of this study as follows:

1. SM was defined as the use of any drug for self-treatment without a valid prescription or guidance from a physician;
2. A physician was defined as an individual authorized to prescribe drugs;
3. Drugs were defined as substances used for the treatment or prevention of illnesses.

### 2.2. Ethical Approval

The study was approved by the Research and Ethics Committee of College of Clinical Pharmacy, King Faisal University, Saudi Arabia (Pharmacy practice, 2017). An informed written consent was sought from all the study participants before conducting the study. A full right to withdraw, confidentiality, and anonymity of the data were guaranteed to all the study participants.

### 2.3. Data Analysis

Data analysis was performed using the Statistical Package for Social Sciences (SPSS) version 25. The results obtained from the analysis were presented in terms of counts and proportions. To compare between different study groups, the Chi-square (χ^2^) test was employed. Descriptive statistics were used to summarize qualitative data in terms of numbers and percentages. Statistical significance was determined by considering a *p*-value of less than 0.05. Any incomplete responses to the questionnaire were excluded from the final analysis to ensure data integrity. It is important to note that in certain questions, where multiple choices were allowed, the total percentage may not always add up to 100%.

## 3. Results

A total of 725 participant (A response rate of 72.5%) were included in the study; of them, 155 never practiced SM. The remaining 570 participants were included in the study; of them, 30 participants did not complete the questionnaire and were excluded from the final data analysis.

Of the remaining 540 participants, 55.6% (*n* = 300) were males and 44.4% (*n* = 240) were females. The mean age of the study respondents was 28 ± 0.6 years. Healthcare professionals, e.g., physicians, nurses, pharmacists, and dentists, represented 15.4% (*n* = 83) of the study population. A total of 69.1% of the participants (*n* = 373) were university graduates, while primary and high school graduates represented 30.9% (*n* = 167) of the population. Among the participants included in the study, a majority of 77.6% (*n* = 419) reported having a good health status, while 22.4% (*n* = 121) indicated suffering from chronic illnesses (Table 1).

SM prevalence among the whole study cohort was 78.6%, with the highest percentage (82%) being among the middle age group (21–40 years) compared to 23% and 44% in the younger and older age groups, respectively. Observed prevalence was higher in the female participants 53.7% (*n* = 161) compared to the males 46.2% (*n* = 111).

Type of education did not affect the attitude of SM practice between different groups, and no statically significant differences were found between medical and non-medical education groups (*p* = 0.374). However, level of education strongly affected its prevalence, with higher incidence among university graduates (*p* < 0.001) Table 2.

**Figure 1:**
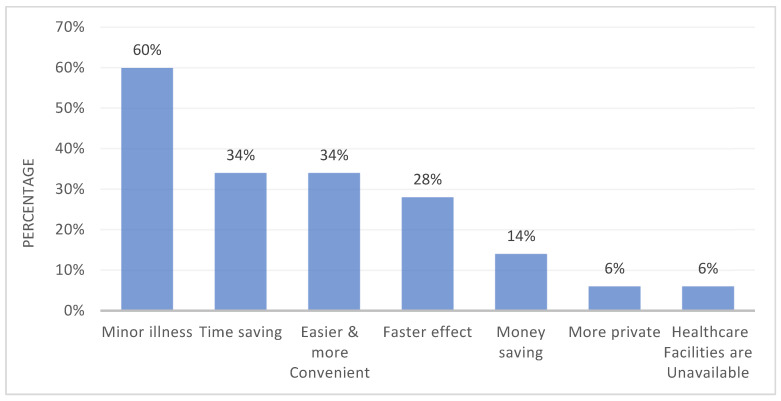
Reasons behind self-medication as reported by the study participants. Data are presented in the form of percentages.

Among healthcare professions, the highest prevalence of SM was found between pharmacists (95%), followed by physicians, nurses, and other medical practitioners (≈70% in all). Study participants reported several reasons behind this practice. Considering their illness to be too trivial for consultation was the main reason in 60% of the study population (*n* = 324), followed by time saving and ease of availability in 34% of the population (*n* = 183) (Figure 1).

Most of the study cohort obtained their self-medicated drugs from private pharmacies (67%), followed by hospital pharmacies (21%), supermarkets (9%), and 3% via the internet. Fear of adverse drug reactions and inappropriate drug usage were the main reasons behind opposing this practice by the study participants.

Of the studied cohort, 109 (20.2%) experienced serious side effects from the consumed drugs. The reported adverse events of the SM drugs are listed in Table 3. More than half the study participants stated that their main source of information about the used drugs is pharmacists (52.8%, *n* = 285) followed by physician assistants (44.1%, *n* = 238). Almost a quarter of the study participants (24.3%, *n* = 131) used their old prescriptions for the same disease as a source for information about the drug. It was also noticed that 39.3% of the participants (*n* = 212) reported the internet as their main source of information (Table 3).

**Figure 2:**
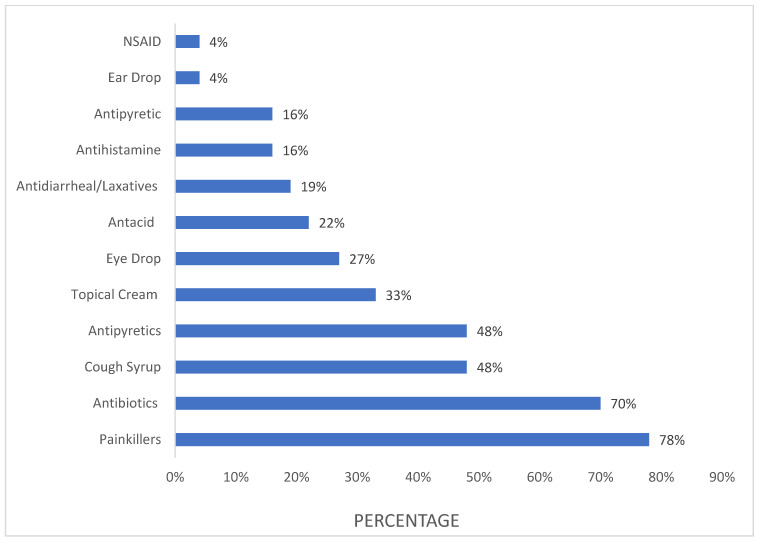
Categories of commonly used drugs in self-medication. Data are presented in the form of percentages (the total percentage is more than 100% as multiple choices were allowed).

Painkillers were the most commonly used class of drugs in SM by the majority of the study participants (78%), followed by antibiotics (70%) and antipyretics and cough syrups (48%). Other drugs used for SM are shown in (Figure 2). The vast majority of the study participants (57.6%) (*n* = 311) read the package insert to acquire information about the drugs.

Among the different indications for SM reported by the participants, headache was the most common (74%) (*n* = 400), followed by fever (72%) (*n* = 389), cough/cold (59%) (*n* = 319), nasal congestion (39%) (*n* = 211), diarrhea (38%) (*n* = 205), muscle pain (33%) (*n* = 178), runny nose (30%) (*n* = 162), heartburn (27%) (*n* = 146), nausea (25%) (*n* = 135), allergy (21%) (114), and mouth ulcer (11%) (*n* = 60) (Table 4).

More than half of the participants (52.9%) reported SM as an acceptable practice, while 11% considered it unacceptable. Only 36% of the participants had a positive attitude towards SM and favored this practice.

## 4. Discussion

Over the past decade, SM has gained widespread recognition in healthcare systems. Extensive research has highlighted both the advantages and disadvantages of SM, depending on the individual’s knowledge about the condition being treated and the extent of the practice.

Despite the KSA’s strict regulations to control the dispensing of drugs without a prescription [38], effectively controlling SM practices has proven to be a challenge. This was attributed to the increased number of private pharmacies and the wide availability of non-prescribed and OTC medications in community pharmacies [39,40]. In accordance with these findings, the prevalence of SM in the current study was found to be 78.6%, with more than 50% of the study population identifying community pharmacies as their primary source for obtaining medications for this practice. These findings align with similar studies conducted in other Gulf countries that share similar national regulations on drug dispensing. For instance, Kuwait reported a SM prevalence of 92% [41], while the United Arab Emirates reported a prevalence of 89% [42]. These findings underscore the significance of SM practices in the region, highlighting the need for effective measures to ensure the safe and responsible use of medications.

Our analysis has also indicated that the highest prevalence of SM is observed among young adults, specifically those aged between 21 and 40 years. These findings align with previous studies, highlighting a higher prevalence of drug misuse and abuse within this age group, primarily due to their limited health literacy and awareness of their own health needs [43,44]. To delve deeper, we recommend exploring the factors contributing to this practice in this age group. This may include factors such as peer pressure, stress, mental health issues, and societal norms. Additionally, economic instability and access to substances can be significant drivers [45,46]. Highlighting the significance of effective health education is also essential in order to elaborate on how it can result in suboptimal decision-making regarding SM. Young adults, due to their limited health knowledge, may not grasp the full extent of the health hazards linked to medications misuse, rendering them more susceptible to engaging in unsafe behaviors [45]. Investigating the interconnections among various factors that promote SM is a valuable step in formulating strategies aimed at mitigating health risks related to drug use among adolescents. Furthermore, these insights can play a pivotal role in raising awareness among adolescents about the potential dangers associated with uninformed and unconsulted drug usage.

Among the various drugs consumed by the study participants, analgesics with antipyretic properties were found to be the most commonly used. This observation aligns with the fact that headache and fever were reported as the most prevalent indications for self-treatment in this cohort. Next, and despite the Saudi adaptation of the WHO antimicrobial resistance global action plan, which aims to increase awareness regarding the misuse of antibiotics in the Kingdom [47], a concerning 70% of the study population reported using antibiotics without a physician’s recommendation. Moreover, among these individuals, 66.9% used antibiotics for a duration of 2 days, while 30.1% admitted to using them for more than 20 days without a proper diagnosis. This practice has the potential to contribute to the spread of multidrug-resistant organisms within the community and is consistent with studies examining antibiotic use among outpatients and the general population in the Kingdom [48,49]. In line with this, international health surveys that have identified Saudi Arabia as having the highest prevalence of multidrug-resistant gram-positive bacteria in the Gulf region [50], which poses a significant public health concern, as the effectiveness of antibiotics in treating bacterial infections diminishes when resistance emerges.

A significant majority of the study population identified pharmacists as their primary source for obtaining misused antimicrobials. These findings are consistent with other studies that have reported 80% of community pharmacists in the Kingdom dispensing antibiotics without prescriptions, with only 1% of pharmacists refusing to sell antibiotics over the counter [51,52]. These alarming results emphasize the immediate need to implement stricter regulations within the healthcare system to prohibit the supply of drugs without prescriptions from community pharmacies. Furthermore, our study revealed that pharmacy clerks and physician assistants were perceived as the easiest way to obtain rapid diagnoses and information about self-medicated drugs. This, again, underscores a significant gap in prescribing and consultation regulations within the Kingdom, emphasizing the importance of introducing non-medical prescribing training for non-medical practitioners. Expanding the prescribing rights to qualified healthcare professionals other than physicians can improve patient care by increasing the availability of qualified healthcare providers. Simultaneously, it would provide patients with faster access to necessary medicines and healthcare services, which was highlighted as a main reason behind SM practices in 34% of our study population.

In many countries, including the United Kingdom (UK) and Scotland, non-medical prescribing has gained considerable traction. It encompasses several healthcare professions, such as nurse prescribers, pharmacist prescribers, and allied health professionals. This model has been pivotal in alleviating the burden on general practitioners, enhancing patient access, and ensuring that individuals receive timely and appropriate treatment from professionals with specialized knowledge. The UK model serves as an exemplary case study for the successful implementation of non-medical prescribing [53,54]. Similarly, in Canada, pharmacists have gained prescribing privileges in some provinces. This model empowers pharmacists to assess and prescribe certain medications, allowing patients to receive immediate advice and treatment for common health conditions. It has been particularly useful in improving access to primary care, especially in underserved areas [55]. We also believe that promoting non-medical prescribing in KSA will be a potential strategy to curb SM practices, given that 34% of our study cohort identified the allure of reducing costs as a significant factor in their decision-making.

Consistent with findings in the literature [56,57], our study revealed a high prevalence of SM among female participants. While some researchers have attributed this phenomenon to physiological factors such as dysmenorrhea or menstrual-associated symptoms, our study observed a higher SM prevalence among women even after adjusting for confounding variables, including chronic and acute disorders.

This higher tendency towards self-care among adult females, as reported by some authors, may reflect their proactive approach to managing their health [58]. However, we believe that cultural factors can also play a significant role; this increased knowledge of drugs and self-care may be a result of cultural norms that encourage them to care for themselves and seek remedies independently [59]. In addition and contrary to the strong evidence linking SM practice to the presence of medications stored at home by family members [58], our findings revealed a higher prevalence of the practice among single individuals. However, this discrepancy can be explained by the greater sense of personal autonomy observed in this group, leading to a lower inclination to seek professional healthcare assistance.

Our study also revealed a positive correlation between education level and the prevalence of SM, which is consistent with findings from existing literature [60,61,62]. These findings support the cultural belief in society that non-medical individuals can acquire knowledge on the proper use of medications, often through learning from older family members or family counsellors. Interestingly, the type of education did not have any significant influence on SM prevalence. Participants with medical education, in particular, did not endorse this practice, attributing their knowledge to a heightened awareness of the adverse effects and toxicities associated with medications. These findings suggest that while education can play a role in increasing the prevalence of SM, it is not solely determined by the type of education received.

Socioeconomic status was one of the main highlighted contributors to increased SM in the literature; low income and low standards of living were reported to be positively associated with increased tendency to self-medicate in many countries [60,61,62]. However, contrary to these findings, our study revealed that higher standards of living and income were directly associated with increased SM practices. On the other hand, self-limiting minor illnesses and time saving were the main reasons behind SM in our cohort; they were also reported as the most prevalent indication for SM in many other studies where “low disease severity” was reported as the main cause followed by the belief that a verbal consultation with a doctor sufficed [63]. This also goes well with our findings wherein participants with a previous illness were more likely to practice SM using remaining medications from other illnesses they suffered from.

While direct-to-consumer advertising (DTCA) of prescription drugs is prohibited in Saudi Arabia and typically directed towards healthcare professionals, the Saudi Food and Drug Authority (SFDA) permits the promotion of OTC drugs directly to the general public. Our study revealed a positive association between DTCPA and SM practices. This finding raises concerns about the regulatory measures surrounding DTCPA, as critics argue that the current rules imposed by the FDA may be too lenient and insufficiently enforced.

DTCPA provides information about medications directly from the drug manufacturers, but it is important to acknowledge that this source of information is susceptible to bias and significant conflicts of interest [64]. The inherent commercial nature of DTCPA raises questions about the accuracy and objectivity of the information provided, potentially leading to incorrect treatment decisions and their subsequent consequences.

Given the potential risks associated with DTCPA, there is a need for stronger regulations and more robust enforcement mechanisms to ensure the integrity and reliability of the information being disseminated to the general public. This includes addressing the issue of bias and conflicts of interest that may arise from the direct involvement of drug manufacturers in promoting their products to consumers.

This study acknowledges several limitations that should be taken into consideration. First, we did not account for the use of medications obtained from the black market, i.e., pharmaceutical substances and prescription medications that are bought, sold, or traded illegally by individuals or electronic platforms. Sharing and secondary distribution from friends and family could also impact the prevalence and patterns of SM. Additionally, as a cross-sectional study, it is important to acknowledge the limitations in establishing causal relationships between variables, as the temporal sequence of events cannot be definitively determined. Therefore, future longitudinal studies are recommended in order to provide a more robust understanding of these relationships.

In conclusion, the alarmingly high prevalence of SM in this study calls for immediate attention. The general population should be better educated and made conscious of the consequences, risks, and side effects of SM. Awareness campaigns may prove to be useful in this matter, allowing the patient to consider the appropriateness of this practice. Individuals in the age group of 21–40 years, females, and those who live alone should be considered priority target populations in the design and implementation of SM awareness projects. Overall, this study provides valuable information that can guide health authorities in combating the widespread practice of SM. Through concerted efforts in regulatory control, it is possible to work towards promoting responsible medication practices and ensuring the well-being of individuals in the Saudi community.

## Figures and Tables

**Table 1:** Distribution of the study participants according to demographic data.

	Self-Medicating Participants (*n* = 540)	Percentage (%)
**Age**		
18–20	65	12.0
21–30	261	48.3
31–40	144	26.7
41–50	55	10.2
>50	15	2.8
**Sex**		
Male	300	55.6
Female	240	44.4
**Education**		
Primary school	5	0.93
High school	162	30.0
University	359	66.5
Master or PhD	14	2.6
**Type of Education**		
Non health education	457	84.6
Health education	83	15.4
**Health status**		
No chronic illnesses	419	77.6
Chronic Illness	121	22.4

**Table 2:** Relation between education level and self-medication practice.

	**Education Level**				**χ^2^**	***p***
	**Primary/High School** **(*n* = 167)**		**University Graduates** **(*n* = 373)**			
	**No.**	**%**	**No.**	**%**		
**How many times in the past year did you treat yourself?**						
I cannot remember	12	7.2	6	1.6	34.675 *	<0.001 *
No	53	31.7	69	18.5		
1	9	5.4	39	10.5		
2	13	7.8	67	18.0		
3	25	15.0	40	10.7		
>4	55	32.9	152	40.8		
	**Type of Education**				**χ^2^**	***p***
	**Non health education** **(*n* = 457)**		**Health education** **(*n* = 83)**			
	**No.**	**%**	**No.**	**%**		
**How many times in the past year did you treat yourself?**						
I cannot remember	16	3.5	2	2.4	5.356	0.374
No	109	23.8	13	15.7		
1	41	9.0	7	8.4		
2	70	15.3	10	12.0		
3	52	11.4	13	15.7		
>4	169	37.0	38	45.8		

χ^2^: Chi square test. *: Statistically significant at *p* ≤ 0.05.

**Table 3:** Main side effects experienced by the study participants and sources of information.

Main Side Effects			
	**Number**	**Percentage with respect to total population (*n* = 540)**	Percentage with respect to individuals experienced SE (*n* = 109) *
GI Upset	50	9.26	45.9
Allergy	47	8.7	43.1
Palpitation	41	7.6	37.6
Headache	18	3.3	16.5
Rash	17	3.2	15.6
Dizziness	16	3.0	14.7
**Sources of information as reported by the study participants**			
	**Number** **(*n* = 540)**	Percentage with respect to total population *	
Pharmacist	285	52.8	
Family	238	44.1	
Internet	212	39.3	
Leaflet	164	30.4	
Friends	138	25.6	
Old prescription	131	24.3	
Book	59	10.9	
Advertisement	25	4.6	

* Total percentage is more than 100% as multiple choices were allowed.

**Table 4:** Distribution of participants according to source of information.

Medical Conditions	Number (*n* = 540)	Percentage (%) with Respect to Total Population *
Headache	400	74
Fever	389	72
Cough/Cold	319	59
Nasal congestion	211	39
Diarrhea/Constipation	205	38
Muscle pain	178	33
Runny nose	162	30
Heartburn/Indigestion	146	27
Nausea	135	25
Allergy	114	21
Vomiting	87	16
Mouth Ulcer	60	11

* Total percentage is more than 100% as multiple choices were allowed.

## Data Availability

Data are contained within the article.
